# Development of a machine learning model to predict intensive care unit bed demand for adult elective surgical patients at a large United Kingdom National Health Service Trust

**DOI:** 10.1016/j.bjao.2025.100513

**Published:** 2026-01-16

**Authors:** Jennifer Hunter, Hrisheekesh Vaidya, Sonya Crowe, Martin Utley, Zella King, Kezhi Li, Steve Harris

**Affiliations:** 1 Institute of Health Informatics, University College London, London, UK; 2 Clinical Operational Research Unit, University College London, London, UK; 3 University College London Hospitals, NHS Foundation Trust, London, UK

**Keywords:** elective, ICU admission, machine learning, perioperative, prediction, surgery

## Abstract

**Background:**

Elective surgical admissions form a growing share of demand for ICU beds, a constrained resource. Capacity planning for these admissions is feasible, but hospitals often lack reliable systems estimating daily elective surgical ICU bed demand before the day of surgery. Comprehensive clinical review of all elective cases is impractical, so planning relies on subjective preassessment processes of variable reliability. This study aimed to develop a machine learning model predicting elective surgical ICU bed demand using electronic health record data to improve on current electronic bed demand estimation at a large UK National Health Service (NHS) Trust.

**Methods:**

Using a retrospective dataset comprising 38 656 elective inpatient surgeries occurring at three sites in a large UK NHS trust between 1 May 2019 and 31 December 2023, we developed two tree-based machine learning models predicting ICU admission after elective surgery: one using only basic, objective clinical data (CoreML) and one using additional preassessment data (FullML). Individual predictions were aggregated to forecast ICU bed demand. Performance was validated retrospectively and prospectively.

**Results:**

At our large UK NHS Trust, in a prospective evaluation, only 71.6% of elective surgical cases admitted to ICU after surgery had an ICU bed electronically requested. In this evaluation, the CoreML model predicting ICU admission at an individual level 1 day before surgery achieved an area under the receiver operator curve of 0.88. It outperformed the current electronic indicator of aggregate elective surgical ICU bed demand 1 day before surgery at two sites handling 72% of inpatient elective surgery (root mean square error, 1.28 *vs* 1.64 at site A; 0.76 *vs* 1.16 at site C). CoreML outperformed FullML in aggregate prediction at all sites in prospective evaluation; however, importantly in retrospective evaluation, the converse was true.

**Conclusions:**

We demonstrate that aggregating individual-level ICU admission predictions for elective surgeries provides a bed demand estimate that improves on the current electronic bed demand indicator 1 day before surgery at two out of three sites conducting the majority of inpatient elective surgery at our large UK NHS Trust. We demonstrate the importance of prospective validation, in which the more parsimonious model was the best performing.

Intensive Care Unit (ICU) beds in the National Health Service (NHS) are a scarce resource.^1^ Elective surgical admissions constitute a significant and growing proportion of ICU bed demand, owing to an increasingly aged and comorbid population undergoing increasingly complex surgeries.^2^^,^^3^ Although the majority of ICU admissions are emergencies, planned elective surgical admissions could be coordinated with expected ICU demand. Estimating elective surgical demand days in advance could allow staff reallocation, earlier rescheduling, or earlier and safer ICU discharge planning. However, hospitals often lack reliable systems providing clinicians managing ICUs or theatres with advance information about planned elective surgical ICU admissions and the likelihood of other surgical cases needing ICU care.^4^

Current ICU bed planning relies on preoperative assessment, during which clinicians request ICU beds for selected cases. Preassessment systems vary between hospitals, but significant limitations are widespread.^5^, ^6^, ^7^ Capacity for anaesthetists to assess patients before surgery is generally limited to those identified as high risk during screening,^8^^,^^9^ and the screening processes are imperfect. Thresholds for requesting postoperative ICU admission vary widely between clinicians.^10^, ^11^, ^12^ Decisions often change on the day of surgery owing to differing clinical opinions^13^ or preassessment failures.^5^ On-the-day cancellations of elective surgery remain common, with a recent UK-wide study^14^ finding that 13.9% of inpatient surgical cases were cancelled on the day of surgery; 10.1% of cancellations were attributable to bed capacity issues, with postoperative ICU bed requirement a strong predictor of cancellation. When ICU beds are unavailable, high-risk patients are either managed on general wards, potentially with worse outcomes,^15^, ^16^, ^17^ or ICU strain increases, which can worsen outcomes across ICU.^18^, ^19^, ^20^ A timely, objective electronic health record (EHR)-based estimate of elective surgical ICU bed demand could reduce day-of-surgery cancellations while ensuring ICU beds are available when needed.

Previous work has suggested that machine learning (ML) models can predict postoperative ICU requirements.^21^, ^22^, ^23^ ML models analyse more variables than traditional statistical risk prediction models^24^^,^^25^ and have outperformed conventional scoring systems in some settings.^18^^,^^26^ ML models can systematically process extensive EHR data and account for prediction uncertainty across large numbers of cases. This is especially useful when considering aggregate predictions for operational planning in a setting where every individual case cannot be assessed in detail by a clinician.

Previous ML models for postoperative ICU admission have focused solely on individual patient predictions or used limited features such as hospital census, day of the week, or surgical case mix.^27^^,^^28^ King and colleagues^29^ demonstrated a novel approach in emergency care: aggregating individual admission probabilities to forecast overall bed demand.

Our aim in this study was to adapt and extend this methodology for elective surgical ICU planning within our busy London Trust, combining individual ML predictions to forecast aggregate bed demand. We aimed to improve on the existing electronic bed demand estimation system at our trust in a prospective evaluation. We hypothesised that a model using a parsimonious set of commonly recorded clinical variables could outperform a model using additional more subjective local data.

## Methods

### Study design and population

Our retrospectively extracted dataset included adult non-obstetric elective inpatient surgeries conducted between 1 May 2019 and 31 December 2023 at three University College London Hospitals (UCLH) Trust sites using data from the Epic EHR system.^30^ We excluded cases with missing operation end times, theatre locations, or operation notes. The three sites handle difficult surgical specialities, and data were pooled on the basis that ML algorithms benefit from larger volumes of data, and that patient features and some surgerical features (such as surgery duration and severity) are likely to be consistent in their utility in predicting ICU admission across surgical sub-specialties.

To evaluate our approach under real-world conditions, we conducted a prospective evaluation, extracting data daily for three weeks (from 26 April to 17 May 2024). This captured cases scheduled within a 3-day operational window, using the same 24-h database update cycle that would be used in clinical deployment.

The COVID-19 pandemic disrupted elective surgery,^31^ creating gaps in our dataset during peak waves, with subsequent periods reflecting altered ICU utilisation patterns.^32^ We partitioned the data chronologically: 70% for training (May 2019–October 2022), 15% for validation (October 2022–May 2023), and 15% for testing (May–December 2023). This temporal split enabled evaluation of model performance across evolving clinical practices and admission criteria.

The study methodology adheres to REFORMS reporting guidelines^33^ (Supplementary material 1.13).

### Ethical approval

The study was conducted as part of the Hyperlocal Demand Forecasts (HYLODE) project, granted approval by the UK Health and Research Authority and Health and Care Research Wales.

### Data source, preprocessing, and datasets

We used Python (version 3.10.10; Python Software Foundation, Wilmington, DE, USA) to extract and analyse the data (see Supplementary material 1.1 for packages used). Extracted features included patient age, surgerical characteristics, medical history, medications, laboratory test results, physiological observations, echocardiograms, and structured preassessment data. We describe feature definitions, transformations, and treatment of missing or impossible data in Supplementary material 1.3.

### Model development

**Fig 1:**
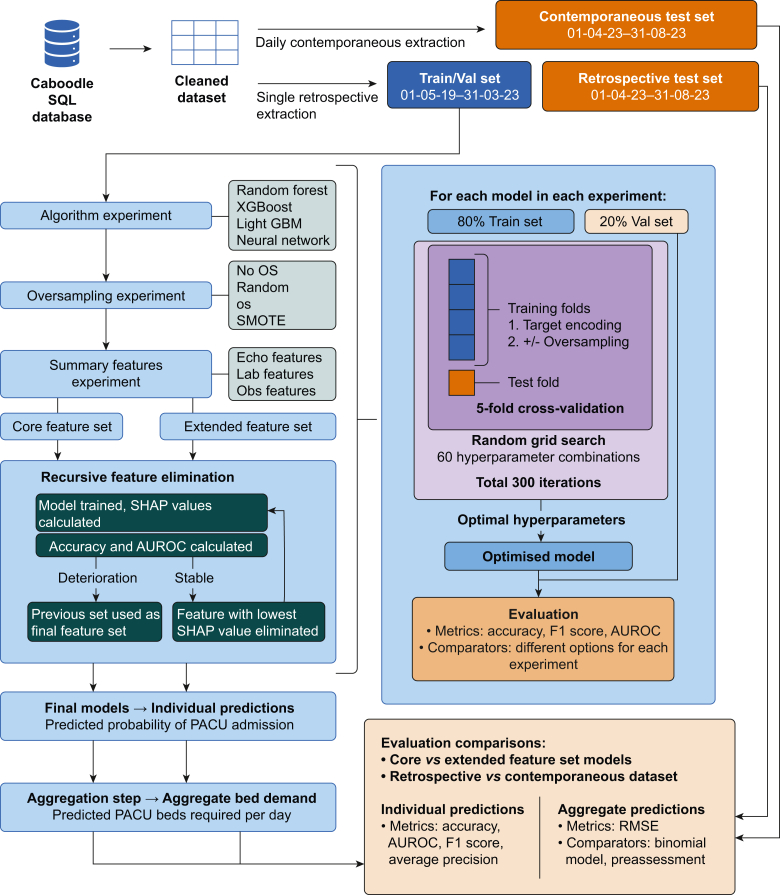
Summary of model development pipeline. AUROC, area under the receiver operating characteristic curve; OS, oversampling; RMSE, root mean square error; SHAP, Shapley additive explanations; SMOTE, synthetic minority oversampling technique; Val set, validation set.

We developed two ML models: a core model (CoreML) using routinely collected clinical data, and an extended model (FullML) using additional preassessment data. As a basis for both models, informed by previous literature on postoperative outcome prediction, we conducted experiments to identify the best performing algorithm, sampling method, and feature transformations using the entire feature set, summarised in Figure 1. For details of algorithms and oversampling methods, see Supplementary material 1.4. We optimised models on the training set using randomised search cross-validation, then evaluated them on the validation set. We performed recursive feature elimination (RFE) using SHAP (SHapley Additive Explanations)^34^ values to identify the minimal feature set required for optimal model performance for each model. For CoreML, we conducted RFE selecting from a parsimonious set of objective features likely to be recorded consistently across hospitals comprising routinely collected clinical measurements (vital signs, laboratory results), surgical characteristics, and basic patient characteristics. For FullML, we conducted RFE selecting from the parsimonious dataset and additional features comprising the postoperative destination selected at case booking, and preassessment data in the form of structured responses to prompts about medical conditions transformed into a numerical value based on severity of conditions affecting a particular organ system.

For each model, we developed two prediction capabilities: individual patient probability of ICU admission and aggregate predictions of daily ICU bed demand. We generated aggregate predictions by combining individual probabilities into a probability distribution for ICU bed requirements. We made these predictions for each of the three hospital sites independently, as the ICUs at these sites operate independently.

### Current booking system and statistical reference model

The current local system for ICU bed planning relies on preoperative assessment, where anaesthetists determine the appropriate postoperative destination for selected cases, either ICU or an inpatient ward. This planned postoperative destination, recorded in the EHR, typically determines postoperative bed allocation unless complications or bed availability issues arise. However, a planned postoperative destination was only recorded before the day of surgery in 51% of cases in our dataset, limiting planning ability. We treated absences of a planned postoperative destination as decisions not to plan ICU admission, the assumption on which the current EHR-based bed booking system operates. In reality, for some cases, this will mean that the appropriate postoperative destination has not been considered before the day of surgery.

We also developed a simple statistical model (StatRef) to predict aggregate ICU bed demand based on day of the week and number of elective surgical cases (Supplementary material 1.2). This provided a computationally efficient reference point for comparing our ML approach.

### Model evaluation

We conducted our evaluation in two phases: retrospective testing and prospective validation. For the retrospective phase, we evaluated both ML models (CoreML and FullML) and the statistical reference model (StatRef) using the held-out test set. For the prospective phase, we implemented a silent running period where predictions were generated daily and available only to the research team.

We assessed performance at both individual and aggregate levels. For individual predictions, we calculated accuracy, sensitivity, specificity, F1 score, area under the receiver operator curve (AUROC), and average precision. For aggregate predictions, we evaluated ICU bed demand forecasts using root mean square error (RMSE) calculated for each hospital site. Confidence intervals for all metrics were bootstrapped where sample size permitted.^35^

To assess temporal stability, we calculated model performance metrics for the retrospective test set as a whole and partitioned by month. For the prospective evaluation, we grouped cases by days until surgery at time of prediction, from 3 days before surgery to the day of surgery. This allowed us to examine how prediction accuracy varied with the forecast horizon.

## Results

### Study population

We analysed 38 656 elective surgical cases, having excluded 23 cases without operation end times and 33 lacking both operation notes and theatre locations. The training and validation sets included 32 857 cases (16.6% ICU admission rate), and the test set contained 5799 cases (16.5% ICU admission rate). Our prospective evaluation dataset comprised 710 cases with an 11.4% ICU admission rate (81 cases). Table 1 presents patient characteristics and surgical characteristics for the retrospective dataset, stratified by postoperative destination. In the retrospective dataset, 80.1% of patients admitted to ICU had an ICU bed requested on either the case booking form or the preassessment form, and 19.8% did not have one requested. In the prospective dataset, only 71.6% of patients admitted to ICU had an ICU bed requested, and 28.4% did not have one requested. 34% of patients who had an ICU bed requested in the prospective dataset were not admitted to ICU.

**Table 1:** Descriptive statistics for patient characteristics and surgical case features. Statistics presented are mean (sd) for continuous variables (∗) and *n* (%) of total ICU or non-ICU patients for categorical variables. GI, gastrointestinal.

Characteristic	ICU patients	Non-ICU patients
	(*n*=6404)	(*n*=32 252)
Sex		
Male	3231 (50.5)	16 044 (49.7)
Female	3173 (49.5)	16 198 (50.2)
Age (years)∗	64.7 (57–76)	57.8 (46–71)
Planned operation duration∗ (min)	233.8 (159.8)	135.8 (71.5)
Primary surgical service		
Urology	1414 (22.1)	10 683 (33.1)
General/colorectal	1185 (18.5)	2828 (8.7)
Thoracic	1017 (15.9)	2080 (6.4)
Gynaecology	837 (13.1)	3262 (10.1)
Head and neck	772 (12.1)	2114 (6.6)
Orthopaedics	461 (7.2)	658 (2.0)
Upper GI	337 (5.3)	387 (1.2)
Other	380 (5.9)	4624 (14.3)
Planned anaesthesia type		
General	4832 (75.5)	26 594 (82.5)
Regional	968 (15.1)	4756 (14.7)
Other	604 (9.4)	902 (2.8)
Priority (booking form)		
Routine	3655 (57.1)	24 732 (76.7)
Cancer pathway	2009 (31.4)	4178 (13.0)
Urgent	416 (6.5)	1976 (6.1)
Unknown	324 (5.1)	1366 (4.2)
Planned postoperative destination (case booking form)		
Inpatient ward bed	1515 (23.7)	16 838 (52.2)
ICU bed	1628 (25.4)	189 (0.6)
Other	200 (3.0)	5473 (16.9)
Unknown	3071 (48.0)	9752 (30.2)
Planned postoperative destination (preassessment)		
Inpatient ward bed	893 (13.9)	19 316 (59.9)
ICU bed	4843 (75.6)	1165 (3.6)
Other	139 (2.2)	5187 (16.1)
Unknown	529 (8.3)	6584 (20.4)
Surgical severity		
Complex	2226 (34.8)	4659 (14.4)
Extra major	1120 (17.5)	6329 (19.6)
Major	1938 (30.3)	9655 (29.9)
Intermediate/minor	834 (13.0)	10 013 (31.0)
Unknown	286 (4.5)	1596 (4.9)

### Model development

The Light Gradient Boosting Machine model without oversampling yielded the best performance. We present performance metrics for alternative algorithms, oversampling methods, and feature transformations in Supplementary material 1.7.

**Fig 2:**
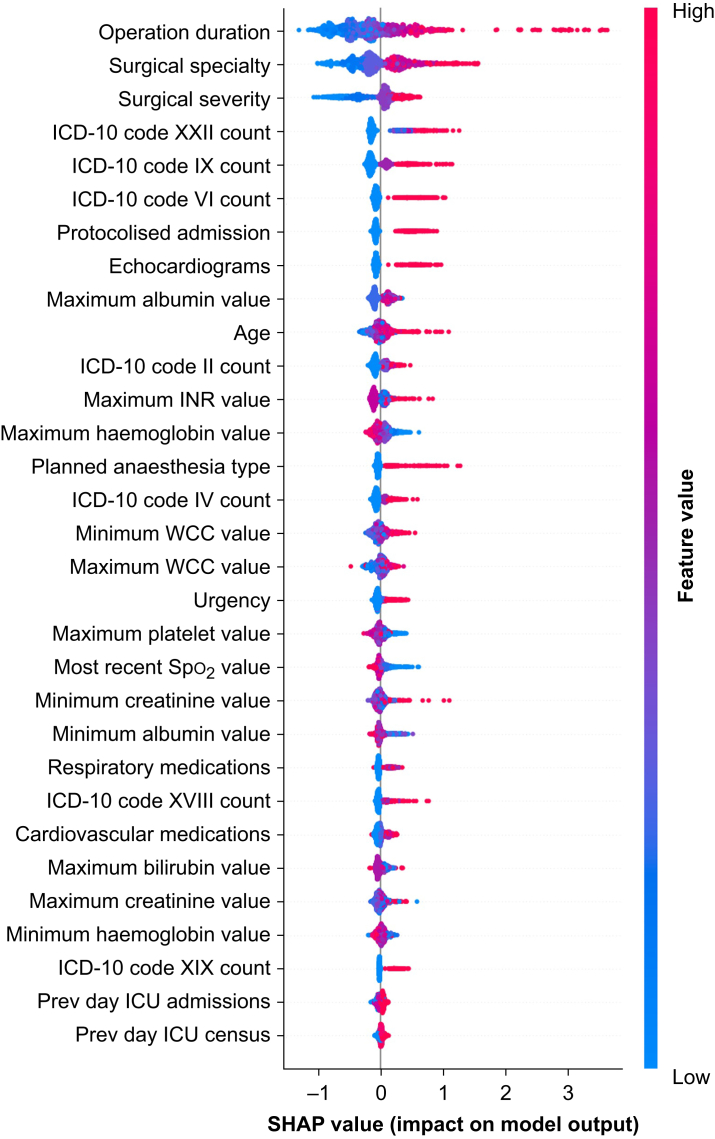
SHAP (SHapley Additive Explanations) force plot for features used in CoreML model. Importance of each feature to the prediction for each of 1000 randomly selected cases in dataset is shown on the X-axis. Features are listed in descending order of average importance. Code XXII: codes for special purpose; code IX: diseases of circulatory system; code VI: disease of nervous system; code II: neoplastic disease; code IV: endocrine, nutritional, and metabolic disease; code XVIII: diseases of the ear and mastoid process; code XIX: injury, poisoning, and certain other consequences of external causes. ICD-10, International Statistical Classification of Diseases and Related Health Problems, 10th Revision; INR, international normalised ratio; SHAP, Shapley additive explanations; Spo_2_, oxygen saturation; WCC, white cell count.

Through RFE, we identified optimal feature sets: 31 features for CoreML and 38 for FullML. We stopped feature elimination when model AUROC or accuracy showed consistent decline (Supplementary material 1.8). Supplementary Tables 1 and 2 provide descriptive statistics for these features in the retrospective and prospective datasets, respectively. Figure 2 illustrates feature importance through a SHAP force plot for CoreML’s 31 features, which shows features in descending order of importance to model predictions. Data availability for each feature before addressing missing values is detailed in Supplementary material 1.9.

### Individual and aggregate prediction performance

**Fig 3:**
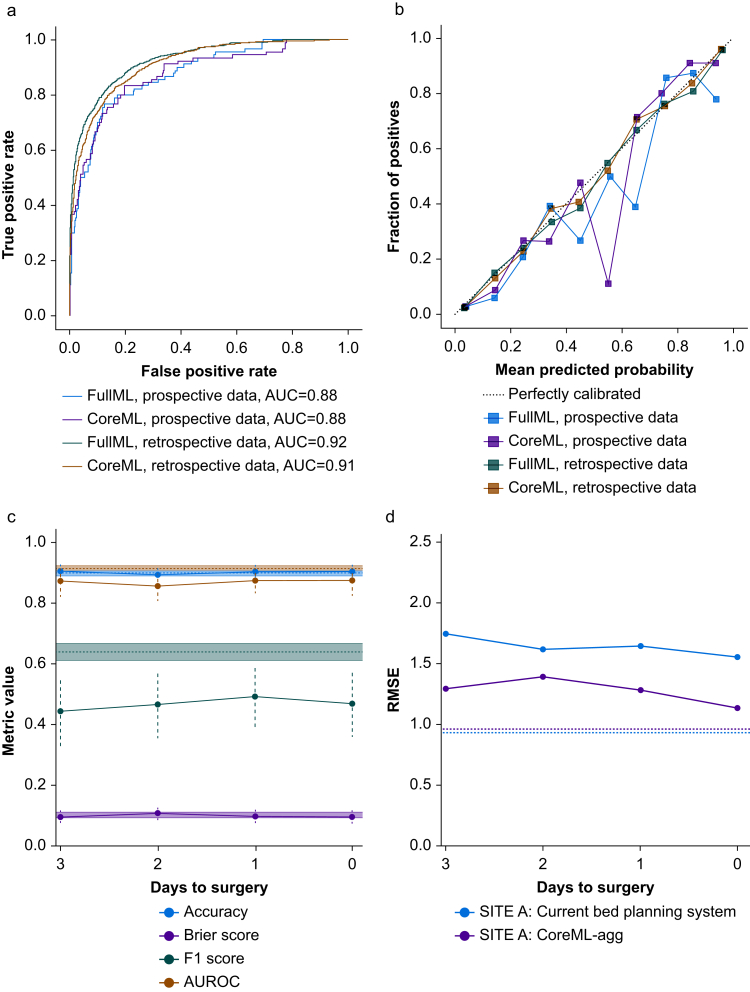
Model evaluation metrics. (a) AUROC plot and (b) calibration plot for CoreML and FullML models evaluated in prospective and retrospective datasets. (c) Change in CoreML model performance metrics accuracy, Brier score, F1 score, and area under the receiver operating characteristic curve (AUROC) as days before surgery at time of prediction increases when evaluated in the prospective dataset. (d) Change in root mean square error (RMSE) of aggregate predictions for site A ICU bed demand from elective inpatient surgery as days before surgery increases for CoreML model and current booking system when evaluated in the prospective dataset. In c and d, dotted line of same colour indicates the metric for same predictor in the retrospective test set, and in c, the shaded area indicates bootstrapped confidence intervals. Confidence intervals could not be calculated for the RMSE owing to small sample sizes.

We evaluated model performance at both individual patient and aggregate demand levels. At the individual level, CoreML and FullML demonstrated good performance in the retrospective test dataset, shown by AUROC (Fig. 3a) and calibration curves (Fig. 3b). In the prospective dataset, both models showed a small deterioration in performance metrics (Table 2), and poorer calibration (Fig. 3b), particularly for predicted probabilities of admission between 0.4 and 0.7. Whereas planned ICU admissions aligned closely with actual admissions in retrospective data, this pattern differed in prospective evaluation as this feature was much less complete. When evaluated on prospective data collected 1 day before surgery, CoreML achieved better accuracy, precision, and Brier scores than the planned postoperative destination recorded in the EHR. Prediction performance at an individual level for CoreML-agg and FullML-agg was stable as the prediction horizon shortened from 3 days before surgery to the day of surgery (Fig. 3c).

**Table 2:** Metrics for model performance in prospective and retrospective evaluation. The column associated with the best performing model/system in each dataset is highlighted in bold for each metric, to illustrate the difference in comparative model performance when evaluated on prospectively and retrospectively collected data. Confidence intervals could not be calculated for the RMSE because of small sample sizes. AUROC, area under the receiver operating characteristic curve; RMSE, root mean square error. CoreML using only objective routinely collected clinical data (vital signs, laboratory results, surgical characteristics, and basic patient characteristics), and FullML incorporating additional pre-assessment information.

Dataset	Prospective test dataset evaluation			Retrospective test dataset evaluation		
Model	CoreML1 day preop	FullML1 day preop	Current bed planning	CoreML1 day preop	FullML1 day preop	Current bed planning
Accuracy	**0.90 (0.88–0.93)**	0.90 (0.88–0.92)	0.90 (0.87–0.92)	0.90 (0.89–0.91)	0.91 (0.90–0.92)	**0.95 (0.94–0.95)**
Precision	**0.74 (0.61–0.86)**	0.65 (0.53–0.77)	0.60 (0.49–0.70)	0.77 (0.74–0.80)	0.80 (0.77–0.83)	**0.86 (0.84–0.88)**
Recall	**0.39 (0.29–0.49)**	0.46 (0.35–0.56)	**0.61 (0.51–0.71)**	0.55 (0.51–0.58)	0.61 (0.58–0.64)	**0.80 (0.78–0.83)**
Brier score	**0.09 (0.07–0.11)**	0.10 (0.08–0.12)	0.10 (0.08–0.13)	0.10 (0.09–0.11)	0.09 (0.08–0.10)	**0.05 (0.05–0.06)**
F1 score	**0.51 (0.40–0.61)**	0.54 (0.44–0.62)	**0.60 (0.52–0.68)**	0.64 (0.61–0.66)	0.69 (0.67–0.72)	**0.83 (0.81–0.85)**
AUROC	**0.88 (0.84–0.92)**	0.88 (0.84–0.91)	-	0.91 (0.90–0.92)	0.92 (0.91–0.93)	-
Average Precision	**0.62 (0.51–0.72)**	0.59 (0.48–0.69)	-	0.74 (0.71–0.77)	0.78 (0.76–0.81)	-
RMSE site A	**1.28**	1.37	1.64	1.10	0.98	**0.96**
RMSE site B	1.46	1.62	**0.97**	1.01	1.01	**0.92**
RMSE site C	**0.76**	0.96	1.16	0.71	0.69	**0.39**

For aggregate demand predictions, we used RMSE to measure prediction error (lower values indicate better accuracy) at each of the three sites separately (values for predictions made 1 day before surgery in Table 2). From 3 days before surgery until the day of surgery, CoreML-agg reduced prediction error compared with use of the EHR-based planned postoperative destination at the largest surgical site, Site A (Fig. 3d) and achieved better or similar RMSEs across this period at Site C (Supplementary Table 4). At Site B, the existing booking system performed best, yielding lower RMSEs than both ML models (Supplementary Table 4).

StatRef, generating only aggregate predictions, showed larger prediction errors than both ML models in the retrospective dataset and higher errors still in the prospective dataset, with RMSE exceeding 4.5 across all sites and time points.

### Temporal stability and data availability

We divided the retrospective test set into eight monthly cohorts to assess temporal stability. CoreML and FullML models maintained consistent performance metrics across all months (Supplementary Fig. 3).

**Fig 4:**
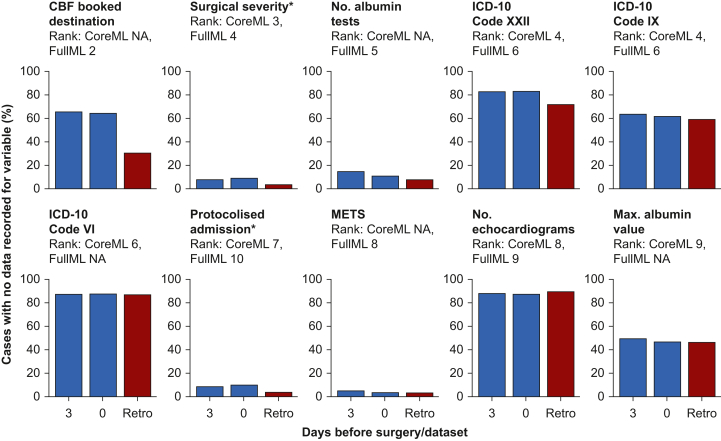
Data completeness for features in the 10 most important features for the CoreML model or the FullML model 3 days before surgery, on the day of surgery, and in the retrospective dataset (Retro). The Y-axis indicates percentage of cases with no data for each variable before handling of missing data. ∗ indicates that this field should be populated, so absence of data always indicates missing data. For all other variables, absence of data indicates that a value has not been recorded for a patient, so may be missing or not applicable. Below the feature name, the feature importance rank of that feature in each model based on the SHAP (SHapley Additive Explanations) values is indicated. Where feature importance is NA, this indicates that feature is not used in that model, having been removed during recursive feature elimination. CBF, Case Booking Form ICD-10, International Statistical Classification of Diseases and Related Health Problems, 10th Revision. METS, Metabolic Equivalent of Task

Figure 4 compares data availability patterns between retrospective and prospective datasets for features appearing in the 10 most important features of either the CoreML or the FullML model. Absence of data does not necessarily represent missing data—for example, if International Statistical Classification of Diseases and Related Health Problems, 10th Revision (ICD-10) coded diagnoses are not recorded for a patient, this may either be because that patient has none of those diagnoses or because they have not yet been recorded in the EHR. Differences in data availability in the retrospective and prospective datasets result from the timing of information becoming available about the patient in the EHR and will impact performance of models trained on retrospective data. Preassessment and booking form data (included only in the FullML model) showed 85–95% completeness in retrospective data but only 45–55% completeness in prospective evaluation. Routine clinical measurements maintained similar completeness levels across both prospective and retrospective datasets, with laboratory results and surgical characteristics showing 90–95% completeness in both evaluations. Features in the top 10 most important features for either model with 100% data completeness in both datasets and therefore not shown in the plot are *planned duration of surgery* (rank 1 for CoreML and FullML), *surgical specialty* (rank 2 for CoreML, rank 3 for FullML), and *age* (rank 10 in core).

## Discussion

### Principal findings

We developed and retrospectively and prospectively internally validated two ML models to predict elective surgical ICU bed demand at a large UK NHS Trust. Our approach combines individual patient predictions with aggregate demand forecasting. Importantly, we report performance in a prospective evaluation, a crucial test rarely undertaken in ML research. The core feature model (CoreML) achieved RMSE improvements in daily bed demand predictions 1 day before surgery of 0.36 at site A and 0.4 at site C, which together conduct 72% of eligible surgical cases. This improvement represents approximately one fewer incorrect prediction every 3 days. FullML achieved RMSE improvements of 0.27 and 0.2 at sites A and C, respectively. At site B, the current bed planning system outperformed both CoreML and FullML 1 day before surgery by 0.49 and 0.65 respectively.

### Comparison with current practice

Current ICU bed planning for elective surgery at the study sites relies on planned postoperative destination recorded in the EHR. This system has significant limitations. In our study period, only 51% of cases had ICU requests recorded before the day of surgery, reflecting the real-world challenges of the limited resources available for preassessment, and busy clinicians making complex decisions. Our institutional audit data show that 13% of ICU admissions after elective surgery were unplanned, highlighting the gap between advance planning and actual need. Some unplanned admissions due to unforeseen intraoperative complications are unavoidable, but our current system’s incomplete coverage suggests room for improvement.

Although preassessment and elective surgical ICU bed planning systems vary between hospitals, unplanned elective surgical ICU admissions are a widespread problem,^36^, ^37^, ^38^, ^39^ as are cancellations related to ICU bed shortages.^14^^,^^40^ Inadequacies in preassessment have been shown to be associated with elective surgery cancellations and postoperative complications.^5^^,^^6^^,^^41^^,^^42^ Use of our approach to predict ICU bed demand could help address these issues without significant additional staff resource. Some hospitals ring-fence ICU beds for elective surgical cases,^43^^,^^44^ but this can be difficult to maintain during busy periods with high volumes of emergency admissions and can lead to surgical admissions competing for beds. A more flexible approach could tailor numbers of ring-fenced beds over a period of days to expected surgical admissions, better matching resources to need.

Our ML approach offers systematic analysis of all cases, considering a wide range of factors that a clinician managing theatres or ICU cannot feasibly assess for every patient. The improvement in RMSE of 0.36–0.40 for aggregate demand predictions across two hospital sites translates to ∼100 additional accurately predicted ICU bed requirements annually. This could significantly impact operational planning. In the third hospital site at which the model was prospectively evaluated, the existing preassessment system outperformed both models. This finding highlights the importance of a prospective local evaluation of ML models in determining where they might be beneficially deployed.

We do not suggest that model outputs be taken as deterministic predictions of admissions, but as with most ML tools developed for use in healthcare, they could augment rather than replace clinical assessment and judgement. Mismatch between model prediction of aggregate demand and planned admissions or available beds could trigger clinical review of cases either booked for ICU after surgery or those not booked but flagged by the model as having a high likelihood of admission. Aggregate demand predictions up to 3 days ahead of surgery could trigger clinician reviews and facilitate earlier interventions such as staff reallocation, review of likelihood and safety of ICU discharges, or rescheduling of cases before the day of surgery.

At an individual patient level, in a system where clinicians are unable to preassess all patients before the day of surgery, model predictions could highlight cases warranting detailed review of postoperative destination planning, supporting more efficient use of limited clinician time and ICU resources.

### Technical performance

Consistent with previous research,^16^^,^^21^^,^^22^^,^^26^ we found tree-based algorithms performed best. However, our approach differs in its parsimony. Through RFE, we developed two models: CoreML using only objective routinely collected clinical data (vital signs, laboratory results, surgical characteristics, and basic patient characteristics), and FullML incorporating additional preassessment information. CoreML’s superior performance in prospective evaluation, despite its smaller feature set, supports its potential generalisability to other centres, as it uses standard clinical data typically available across different healthcare systems. This finding is crucial—retrospective evaluation alone would have favoured the more complex FullML model, potentially leading to the incorrect conclusion that our approach was not viable for clinical implementation. The superior performance of CoreML aligns with statistical learning theory—simpler models often show better bias-variance trade-off in real-world applications, and is also likely attributable in part to data missingness in live data which we describe.

Importantly, we are not comparing the performance of our model against a clinician’s judgement on need for postoperative ICU admission for each patient, but against the system for planning elective surgical ICU admissions at our trust, which acts as a realistic benchmark for the proposed use case. Although at an individual patient level a clinician prediction of need for ICU after elective surgery is likely to be superior to an ML prediction, clinician review is not possible for all patients before the day of surgery, whereas an ML prediction of ICU admission using routinely collected data can be automated and provided for all patients. The strength of this approach lies in automation and aggregate predictions across large patient volumes.

### Methodological considerations

Our study addresses critical challenges in healthcare ML implementation.^45^ Most ML models are evaluated only on retrospective data^46^, which may not reflect deployment conditions. Studies using live data often show worse performance than retrospective evaluations.^47^^,^^48^ We include both retrospective and prospective evaluation in assessing real-world utility. Our prospective evaluation revealed important differences: whereas the current ICU admission planning system showed apparent superior performance in retrospectively extracted data, CoreML demonstrated better real-world prediction capability. Quantification of data availability in the retrospectively and prospectively extracted datasets showed that preassessment features included in the FullML model were less complete in live data, suggesting that these data were missing in the live dataset and were made available retrospectively. This contributed to the greater drop in performance of FullML in prospective evaluation. This highlights the importance of analysis of and evaluation on live data in application of ML to healthcare data.

We demonstrated robust temporal stability of model performance with both models maintaining consistent performance across 8 months of retrospective data. Performance differences in prospective testing likely reflect real-world data completeness patterns rather than temporal drift. This understanding of data quality variation between training and deployment conditions is crucial for operational implementation.

CoreML’s superior performance addresses another key implementation challenge: EHR data availability.^49^ While UK NHS trusts use different EHR systems, CoreML’s reliance on commonly recorded clinical data suggests potential for wider application.

Our study highlights critical considerations for ML implementation using EHR data. ML models are typically trained on large retrospective datasets, with performance reported on held-out test data. However, real-world implementation must account for differences in how EHR databases are populated and updated in real time. Understanding these differences is crucial for developing robust, deployable systems. Our approach demonstrates that models can maintain performance despite these challenges when designed with real-world constraints in mind.

### Limitations and future work

Although its parsimonious feature set provides potential for CoreML to generalise to other settings, local variations in postoperative ICU admission practices for elective surgical cases may limit CoreML’s generalisability. Data availability and missingness patterns are also likely to differ significantly between healthcare systems. Instead, we suggest use of the approach we describe of using local data to develop ML models predicting ICU admission, and aggregating predictions to estimate ICU bed demand from elective surgery, which demonstrates superior performance to the current ICU bed planning system in our trust. We also emphasise the importance of local analysis of live data, and prospective evaluation of ML models in healthcare.

The potential for aggregate demand prediction models to deliver improvements in a given hospital will vary widely depending on the current bed planning system, data availability, and how the EHR is used. Given the EHR indicators of planned postoperative destination are poorly complete until the day of surgery at our trust, there is significant scope for improvement. Our model performance is evaluated against EHR indicators of planned ICU admission; in reality, some ICU admission decisions may have been made but not recorded in the EHR. At our trust the EHR indicators of postoperative destination are relied on for operational decisions, making our assumptions fair for evaluation of a model for operational purposes. This may not be the case at other centres. Additionally, the performance of EHR-based predictions will be dependent on when data are available in the EHR, and is likely to be better in settings where the majority of data are recorded in the EHR by the point at which a prediction is intended to be used.

Future work will include a multicentre analysis comparing performance at each centre of a single model trained using multicentre data to predict ICU admission against the performance of models trained on local data separately for each centre. Further work will focus on clinical implementation, particularly prediction delivery methods. We will incorporate SHAP values to explain individual predictions and provide data completeness metrics to indicate prediction reliability. Clinical user evaluation and patient/public involvement will be essential to ensure safe, sustainable deployment.

## Conclusions

We demonstrate a reproducible methodology for predicting ICU bed demand after elective surgery using ML. Our approach combines four key elements: development of models using routinely collected clinical data, identification of a minimal but robust feature set, aggregation of individual predictions for operational forecasting, and thorough evaluation under real-world conditions. At two major hospital sites, this methodology matched or exceeded current booking systems for predicting ICU bed demand in the 3 days before surgery, while maintaining performance during prospective validation testing. The parsimonious nature of our core model, using only commonly recorded clinical variables, suggests potential for wider implementation. This approach could both improve operational efficiency and systematically identify cases requiring detailed clinical review of postoperative destination planning.

## Authors’ contributions

Performed the literature search, generated the figures: JH

Conducted the modelling and evaluation, co-wrote the paper: JH, HV

Contributed to the modelling design: HV

Contributed to the modelling design and drafting: ZK, SC, MU, KL

Conceived the study, secured funding, contributed to model evaluation and drafting: SH

## Data and materials availability

The datasets analysed in this research are not publicly available. Researchers wishing to validate or replicate this work using the same datasets would need to be approved for research collaborations with University College London Hospitals NHS Foundation Trust, and to secure appropriate permissions from the UCLH/UCL Joint Research Office. Researchers who meet these requirements can contact the corresponding author for further information about access to the datasets.

## Funding

National Institute for Health Research (Artificial Intelligence, Digitally adapted, hyper-local real-time bed forecasting to manage flow for NHS wards, AI AWARD01786); NHSX; 10.13039/501100000272National Institute for Health and Care Research University College London Hospitals Biomedical Research Centre.

## Declaration of interest

The authors have no conflicts of interests to declare.
