# Supplementary material for: Development of a machine learning model to predict intensive care unit bed demand for adult elective surgical patients at a large United Kingdom National Health Service Trust
Source: BJA Open. 2026 Jan 16;17:100513. doi: 10.1016/j.bjao.2025.100513 (PMC12830247; doi:10.1016/j.bjao.2025.100513)
Supplement: Multimedia component 1 [file mmc1.docx]

# Supplementary Material

## 1.1 Computing Infrastructure

A list of Python packages and versions used is given below:

| IPython: 7.29.0  argparse: 1.1  arrow: 1.2.1  astunparse: 1.6.3  attr: 23.1.0  backcall: 0.2.0  category encoders: 2.6.1  certifi: 2019.11.28  cffi: 1.15.1  chardet: 3.0.4  charset normalizer: 2.0.12  click: 8.1.3  cloudpickle: 2.0.0  colorama: 0.4.6  comm: 0.1.3  csv: 1.0  ctypes: 1.1.0  cycler: 0.10.0  datatable: 1.0.0  dateutil: 2.8.2  debugpy: 1.6.7  decimal: 1.70  decorator: 5.1.1  defusedxml: 0.7.1  dill: 0.3.6  distutils: 3.9.5  dns: 2.6.1  entrypoints: 0.4  environs: 9.3.5  fastapi: 0.70.0  filelock: 3.13.1  flatbuffers: 24.3.25  google.protobuf: 3.20.3  greenlet: 2.0.2  h5py: 3.12.1  http.server: 0.6  hylib: 0.5.0  idna: 2.8  imblearn: 0.10.1 | ipaddress: 1.0  ipykernel: 6.22.0  ipywidgets: 8.0.6  jax: 0.4.30  jedi: 0.18.2  joblib: 1.2.0  json: 2.0.9  jupyter client: 8.2.0  jupyter core: 5.3.0  keras: 3.6.0  kiwisolver: 1.4.4  lightgbm: 3.3.2  llvmlite: 0.43.0  logging: 0.5.1.2  marshmallow: 3.19.0  matplotlib inline: 0.1.6  matplotlib: 3.7.1 ml  dtypes: 0.4.1  mlflow: 1.26.1  mlflow.utils.gorilla: 0.3.0  mpmath: 1.3.0  numba: 0.60.0  numexpr: 2.8.4  numpy: 1.26.4  opt einsum: v3.3.0  optree: 0.13.0  packaging: 23.1  pandas: 1.4.4  parso: 0.8.3  patsy: 0.5.3  pexpect: 4.8.0  pickleshare: 0.7.5  platform: 1.0.8  platformdirs: 4.0.0  prompt toolkit: 3.0.38  psutil: 5.8.0  psycopg2: 2.9.5  ptyprocess: 0.7.0  pyarrow: 6.0.1 | pydantic: 1.10.2  pydevd: 2.9.5  pygments: 2.15.1  pyparsing: 3.0.9  pytz: 2023.3  re: 2.2.1  requests: 2.28.1  scikeras: 0.11.0  scipy: 1.13.1  seaborn: 0.11.2  setuptools: 67.7.2  shap: 0.41.0  six: 1.14.0  sklearn: 1.2.2  sniffio: 1.3.0  socketserver: 0.4  sqlalchemy: 1.4.43  starlette: 0.16.0  statsmodels: 0.13.5  sympy: 1.11.1  tensorflow: 2.17.0  tensorflow.keras: 3.6.0  threadpoolctl: 3.1.0  torch: 1.10.1+cpu  tqdm: 4.65.0  tqdm.cli: 4.65.0  traitlets: 5.9.0  ujson: 5.7.0  urllib.request: 3.9  urllib3: 1.26.15  wcwidth: 0.2.6  wrapt: 1.14.1  xgboost: 1.5.2  xmlrpc.client: 3.9  yaml: 6.0  zlib: 1.0  zmq: 25.0.2  zmq.sugar: 25.0.2 |
| --- | --- | --- |

## 1.2 Binomial Prediction Model

A binomial prediction model was trained on a dataset including surgical case occurring between the 15/09/2020 and 23/05/23. The start date for inclusion in the dataset used for training the binomial model is later than that used for the dataset used to train the ML model as the data regarding which hospital wards were used as surgical wards was not available earlier than 15/09/2020. For each date in this dataset, the number of surgical cases meeting inclusion criteria, the number of cases meeting inclusion criteria going to PACU postoperatively, and the total number of patients on the surgical wards on that day, were extracted. The total number of patients was scaled using a MinMaxScaler() function, meaning all the values were transformed to take a value between 0 and 1. The dataset was split into an 80% training set, and a 20% test set. The predictor variables used in the binomial model were the day of the week, and the census of the surgical wards. Month and season were also trialed as predictor variables, but were found not to be significant predictors of ICU admission rates. Three separate models were trained for each of the three UCLH Trust sites, as the surgical census predictor variables has a very different relationship with the proportion of cases admitted to ICU in each site.

## 1.3 Data Transformations

Details of feature definitions, sources, transformations and treatment of missing or impossible values are shown in sheet feature definitions in supplementary material.xlsx, along with how missing or impossible values for each field were treated.

## 1.4 ML Algorithms and Oversampling Techniques

### 1.4.1 ML Algorithms

The majority of literature describing ML algorithms predicting postoperative outcomes including ICU admission report that tree based algorithms are the best performing, so three tree based algorithms were trialled: Light Gradient Boosting Machine (LGBM), eXtreme Gradient Boosting (XGBoost), and random forest (RF). These were compared with a simple three layer fully connected deep neural network (NN) with optional drop out and batch normalisation layers which were varied as hyperparameters. This type of NN is used widely used as an ML benchmark. Hyperparameter combinations evaluated using a random grid search are detailed below for each model, and are based on published literature on appropriate hyperparameter combinations for each of these models.

### ***LGBM Model***

For the LGBM model a logistic loss function was used. The parameters used to perform a randomised grid search for hyperparameter tuning were:

- Number of estimators: ([100, 150, 200])
- Learning rate: ([0.01, 0.05, 0.1, 0.5])
- Maximum depth: ([5, 10, 15, 20, 25, 30])
- Number of leaves: ([10, 20, 40, 60])
- Alpha: ([0.001, 0.01, 0.1, 1, 10, 100])
- Lambda: ([0.001, 0.01, 0.1, 1, 10, 100])
- Minimum child samples: ([10, 20, 30])
- Columns sampled by tree: ([0.3, 0.5, 0.8, 1])
- Extra trees: ([True, False])

The hyperparameters used by the both of the final LGBM models trained on the training and testing sets from the retrospective dataset were: number of estimators: 200, learning rate: 0.05, maximum depth: 30, number of leaves: 60, alpha: 0.1, lambda: 1, minimum child samples: 20, columns sampled by tree: 0.3, extra trees: False.

### ***XGBoost Model***

For the XGBoost model a logistic loss function was used. The parameters used to perform a randomised grid search for hyperparameter tuning were:

- Number of estimators: ([50, 100, 150, 200])
- Learning rate: ([0.001, 0.01, 0.1, 0.3])
- Maximum depth: ([3, 5, 8, 10, 12])
- Minimum child weight: ([5, 7, 10])
- Gamma: ([0.5, 1, 5, 10, 15, 20])
- Alpha: ([0.001, 0.01, 0.1, 1, 10, 100])
- Lambda: ([0.001, 0.01, 0.1, 1, 10, 100])
- Subsampling proportions: ([0.5, 0.7, 0.9])
- Columns sampled by tree: ([0.5, 0.7, 0.9])
- Scale positive weight: ([1, 5, 10])

### ***Random Forest Model***

For the Random Forest model the Gini function was used as the optimisation metric. The parameters used to perform a randomised grid search for hyperparameter tuning were:

- Number of estimators: ([100, 200, 400, 600, 800])
- Maximum depth: ([10, 20, 30, 40, 60, 80, 100])
- Maximum features: ([’auto’, ’sqrt’])
- Minimum samples per leaf: ([1, 2, 4, 8])
- Minimum samples per split: ([2 ,5, 10]) – Bootstrap: ([True, False])

### ***Feed Forward Neural Network***

For the fully connected neural network model the logistic loss function was used. The basic architecture was a three layers neural network with additional option batch normalisaiton and dropout layers, the inclusion of which was treated as a hyperparameter. The parameters used to perform a randomised grid search for hyperparameter tuning were:

- Neurons in layer 1: ([16, 32, 64])
- Neurons in layer 2: ([32, 64,128])
- Neurons in layer 1: ([16, 32, 64])
- Dropout layer 1: ([True, False])
- Dropout layer 2: ([True, False])
- Dropout layer 3: ([True, False])
- Dropout probability: ([0.1, 0.2, 0.3])
- Batch normalisation layer 1: ([True, False])
- Batch normalisation layer 2: ([True, False])
- Batch normalisation layer 3: ([True, False])
- Learning rate: ([0.001, 0.01, 0.05, 0.1])

### 1.4.2 Oversampling Methods

Oversampling methods have been reported to improve performance in some settings when a minority class is being predicted, as is the case for our admission ICU prediction task. We trialled random oversampling (ROS) and Synthetic Minority Oversampling Technique (SMOTE) to determine whether these improved predictive performance. We trialled oversampling to proportions of 0.4, 0.6, and 0.8 of the majority class.

### 1.4.3 Hyperparameter Tuning

To determine optimal hyperparameter combinations, a random grid search of 60 combinations of hyperparameters with 5-fold cross validation was performed using the training set. A model using the optimal hyperparameter combination was trained on the whole training set and evaluated in the validation set for each experiment. When oversampling methods were used, oversampling was performed individually on each set of evaluation folds during random grid search cross-validation, with no oversampling performed on the holdout fold.

## 1.5 Descriptive Statistics for Full List of Features Used in FullML Model in Retrospectively Extracted Dataset

| **Features** | **ICU Patients**  **(n = 6,404)** | | **Non-ICU Patients**  **(n = 32,252)** | | |  |
| --- | --- | --- | --- | --- | --- | --- |
|  | Mean  (N) | SD  (%) | Mean  (N) | SD  (%) | | |
| **Sex***  Male | (3,231) | (50.5) | (16,044) | (49.7) | | |
| Female | (3,173) | (49.5) | (16,198) | (50.2) | | |
| Unknown | (0) | (0) | (10) | (0.0) | | |
| **Age** | 64.72 | 15.25 | 57.76 | 17.03 | | |
| *Features of surgery* |  |  |  |  | | |
| **Planned operation duration (mins)** | 233.77 | 159.83 | 135.76 | 71.53 | | |
| **Primary surgical service** | | | | | |  |
| Urology | (1,415) | (22.1) | (10,683) | (33.1) | | |
| Thoracic | (1,017) | (15.9) | (2,080) | (6.4) | | |
| Gynecology | (837) | (13.1) | (3,262 | (10.1) | | |
| Head and neck | (772) | (12.1) | (2,114) | (6.6) | | |
| General | (674) | (10.5) | (2,170) | (6.7) | | |
| Other | (1,689) | (26.3) | (11,943) | (37.0) | | |
| **Planned anaesthesia type** | | | | | |  |
| General | (4,832) | (75.5) | (26,594) | (82.5) | | |
| Regional | (968) | (15.1) | (4,756) | (14.7) | | |
| Other | (604) | (9.4) | (902) | (2.8) | | |
| **Priority (booking form)** | | | | | |  |
| Routine | (3,655) | (57.1) | (24,732) | (76.7) | | |
| Urgent | (416) | (6.5) | (1,976) | (6.1) | | |
| Cancer pathway | (2,009) | (31.4) | (4,178) | (13.0) | | |
| Unknown | (324) | (5.1) | (1,366) | (4.2) | | |
| **Surgical severity** | | | | | |  |
| Complex | (2,226) | (34.8) | (4,659) | (14.4) | | |
| Extra major | (1,120) | (17.5) | (6,329) | (19.6) | | |
| Major | (1,938) | (30.3) | (9,655) | (29.9) | | |
| Intermediate | (628) | (9.8) | (6,037) | (18.7) | | |
| Minor | (206) | (3.2) | (3,967) | (12.3) | | |
| Unknown | (286) | (3.2) | (1,596) | (4.9) | | |
| **Urgency (preassessment form)** | | | | | |  |
| Expedited | (521) | (8.1) | (1,745) | (5.4) | | |
| Elective | (5,883) | (91.9) | (30,507) | (94.6) | | |
| Unknown | (0) | (0) | (0) | (0) | | |
| **Protocolised admission** |  |  |  |  | | |
| Yes | (2,078) | (32.4) | (2,660) | (8.2) | | |
| No | (4,326) | (67.6) | (29,592) | (91.8) | | |
| *Selected Postoperative Destinations* | | | | | |  |
| **Planned postoperative destination (booking form)** | | | | | |  |
| Inpatient ward bed | (1,515) | (23.7) | (16,838) | (52.2) | | |
| PACU bed | (1,628) | (25.4) | (189) | (0.6) | | |
| Day surgery bed | (173) | (2.7) | (5,042) | (15.6) | | |
| Other | (17) | (0.3) | (431) | (1.3) | | |
| Unknown | (3,071) | (48.0) | (9,752) | (30.2) | | |
| **PACU bed requested (preassessment form)** | | | | | |  |
| Yes | (4843) | (75.6) | (1165) | (3.6) |  |  |
| No | (1561) | (24.4) | (31087) | (96.4) |  |  |
| *Investigations* |  |  |  |  |  |  |
| **Abnormal creatinine measurements** | 0.90 | 3.97 | 0.32 | 2.13 |  |  |
| **Abnormal WCC measurements** | 0.97 | 3.50 | 0.42 | 2.35 |  |  |
| **Abnormal platelet measurements** | 0.85 | 3.29 | 0.33 | 2.27 |  |  |
| **Abnormal haemoglobin measurements** | 1.59 | 6.13 | 0.55 | 3.38 |  |  |
| **WCC measurements** | 3.43 | 6.98 | 1.84 | 3.91 |  |  |
| **Albumin measurements** | 2.76 | 6.28 | 1.12 | 3.54 |  |  |
| **INR measurements** | 1.30 | 2.82 | 0.70 | 1.47 |  |  |
| **Number of echocardiograms** | 0.21 | 0.41 | 0.08 | 0.27 |  |  |
| *Preassessment Patient History* |  |  |  |  |  |  |
| **Cardiac conditions** | 0.46 | 1.07 | 0.21 | 0.66 |  |  |
| **Respiratory conditions** | 0.40 | 1.11 | 0.16 | 0.67 |  |  |
| **Renal conditions** | 0.34 | 1.31 | 0.14 | 0.76 |  |  |
| **METS classification** | 3.11 | 2.75 | 3.64 | 2.79 |  |  |
| **Anaesthetic alert** | 0.53 | 0.67 | 0.35 | 0.57 |  |  |
| **Cardiovascular medications** | 1.57 | 2.12 | 1.01 | 1.75 |  |  |
| **Endocrine medications** | 0.72 | 1.47 | 0.52 | 1.26 |  |  |
| *ICD-10 Code Counts* | | | | |  |  |
| **II: Cancer conditions** | 1.35 | 1.54 | 0.53 | 0.89 |  |  |
| **IV: Endocrine conditions** | 1.22 | 1.72 | 0.49 | 0.92 |  |  |
| **IX: Cardiovascular conditions** | 1.39 | 1.95 | 0.47 | 0.92 |  |  |
| **VI: Central nervous system conditions** | 0.30 | 0.71 | 0.14 | 0.47 |  |  |
| **XIX: Injury and poisoning conditions** | 0.41 | 1.00 | 0.13 | 0.50 |  |  |
| **XVIII: Other conditions** | 1.25 | 2.35 | 0.37 | 0.99 |  |  |
| **XXII: Other specialized codes** | 2.21 | 3.36 | 0.46 | 1.48 |  |  |
| *ICU demand metrics* |  |  |  |  |  |  |
| **ICU admissions previous day**  Low demand | (1,024) | (16.0) | (6,735) | (20.9) |  |  |
| Normal demand | (3,437) | (15,414) | (53.7) | (47.8) |  |  |
| High demand | (1,943) | (30.3) | (10,103) | (31.3) |  |  |
| **ICU census previous day**  Low demand | (1,471) | (23.0) | (6,695) | (20.8) |  |  |
| Normal demand | (3,370 ) | (52.6) | (16,073) | (49.8) |  |  |
| High demand | (1,563) | (24.4) | (9,484) | (29.4) |  |  |

Table S1: Descriptive statistics for features included in model after feature selection process in the training set. Statistics presented are mean and standard deviation (SD) for continuous variables, and count and percentage of total ICU or non-ICU patients for categorical variables. *Sex was removed during RFE and is not a feature in the final model, but is included here for descriptive purposes. WCC: white cell count, INR: international normalised ratio, METs: metabolic equivalence tasks

## 1.6 Descriptive Statistics for Full List of Features Used in FullML Model in Prospectively Extracted Dataset

Note, the descriptive table below describes cases in the prospectively collected dataset that occurred, describing the data collected on the day of surgery. Additional cases are included in this dataset which were ultimately cancelled, but are not described in this dataset. They are included for the purposes of evaluation as this is necessary to give a realistic indication of performance on live data.

| **Features** | | **ICU Patients**  **(n = 81)** | | | | **Non-ICU Patients**  **(n = 629)** | | |
| --- | --- | --- | --- | --- | --- | --- | --- | --- |
|  |  | Mean  (N) | | SD  (%) | | Mean  (N) | | SD  (%) |
| **Sex*** | |  | |  | |  | |  |
| Male | | (40) | | (50.6) | | (330) | | (52.5) |
| Female | | (40) | | (49.4) | | (299) | | (47.5) |
| **Age** | | 59.6 | | 16.6 | | 56.9 | | 16.1 |
| *Features of surgery* | |  | |  | |  | |  |
| **Planned operation duration (mins)** | 258.7 | | 148.6 | | 149.8 | | | 73.0 |
| **Primary surgical service** | | | | | | | | |
| Urology | (14) | | (17.3) | | (165) | | | (26.2) |
| Thoracic | (13) | | (16.0) | | (30) | | | (4.8) |
| Gynecology | (13) | | (16.0) | | (68) | | | (10.8) |
| Head and neck | (13) | | (16.0) | | (49) | | | (7.8) |
| General | (11) | | (13.6) | | (46) | | | (7.3) |
| Other | (9) | | (11.1) | | (271) | | | (43.1) |
| **Planned anaesthesia type** | | | | | | | | |
| General | (72) | | (88.9) | | (503) | | | (80.0) |
| Regional | (2) | | (2.5) | | (76) | | | (12.1) |
| Other | (7) | | (8.6) | | (50) | | | (7.9) |
| **Priority (booking form)** | | | | | | | | |
| Routine | (57) | | (70.4) | | (519) | | | (82.5) |
| Cancer pathway/Urgent | (13) | | (16.0) | | (46) | | | (7.3) |
| Unknown | (11) | | (13.6) | | (64) | | | (10.2) |
| **Planned postoperative destination (booking form)** | | | | | | | | |
| Inpatient ward bed | (10) | | (12.3) | | (151) | | | (24.0) |
| PACU bed | (3) | | (3.7) | | (5) | | | (0.8) |
| Day surgery bed/Other | (0) | | (0) | | (30) | | | (4.8) |
| Unknown | (68) | | (84.0) | | (443) | | | (70.4) |
| **Surgical severity** | | | | | | | | |
| Complex | (31) | | (38.33) | | (84) | | | (13.4) |
| Extra major | (15) | | (18.5) | | (158) | | | (25.1) |
| Major | | (25) | | (30.9) | | (207) | (32.9) | |
| Intermediate | | (6) | | (7.4) | | (72) | (11.4) | |
| Minor | | (0) | | (0) | | (35) | (5.6) | |
| Unknown | | (4) | | (4.9) | | (72) | (11.4) | |
| **Urgency (preassessment form)** | | | | | | | | |
| Immediate | | (0) | | (0) | | (0) | (0) | |
| Urgent | | (0) | | (0) | | (3) | (0.5) | |
| Expedited | | (3) | | (3.7) | | (11) | (1.7) | |
| Elective | | (20) | | (24.7) | | (150) | (23.8) | |
| Unknown | | (58) | | (71.6) | | (465) | (73.9) | |
| **Protocolised admission** | |  | |  | |  |  | |
| Yes | | (28) | | (34.6) | | (42) | (6.7) | |
| No | | (49) | | (60.5) | | (515) | (81.9) | |
| Unknown | | (4) | | (4.9) | | (72) | (11.4) | |
| *Selected Postoperative Destinations* | |  | |  | |  |  | |
| **Planned postoperative destination (booking form)** | | | | | | | | |
| Inpatient ward bed | | (10) | | (12.3) | | (151) | (24.0) | |
| PACU bed | | (3) | | (3.7) | | (5) | (0.8) | |
| Day surgery bed | | (0) | | (0) | | (30) | (4.8) | |
| Other | | (68) | | (84.0) | | (443) | (70.4) | |
| **PACU bed requested (preassessment form)** | | | | | | | | |
| Yes | | 58 | | (71.6) | | 30 | (4.8) | |
| No | | 23 | | (28.4) | | 599 | (95.2) | |
| *Investigations* | |  | |  | |  |  | |
| **Abnormal creatinine measurements** | | 0.73 | | 2.06 | | 0.30 | x 1.10 | |
| **Abnormal WCC measurements** | | 0.62 | | 1.26 | | 0.35 | 1.12 | |
| **Abnormal platelet measurements** | | 0.65 | | 1.98 | | 0.22 | 0.93 | |
| **Abnormal haemoglobin measurements** | | 0.94 | | 2.79 | | 0.50 | 2.17 | |
| **WCC measurements** | | 2.86 | | 3.60 | | 1.88 | 2.53 | |
| **Albumin measurements** | | 2.33 | | 3.23 | | 1.09 | 2.18 | |
| **INR measurements** | | 1.05 | | 1.12 | | 0.71 | 0.95 | |
| **Number of echocardiograms** | | 1.0 | | 0.0 | | 1.0 | 0.0 | |
| *Preassessment Patient History* | |  | |  | |  |  | |
| **Cardiac conditions** | | 0.32 | | 0.79 | | 0.22 | 0.77 | |
| **Respiratory conditions** | | 0.30 | | 0.89 | | 0.13 | 0.13 | |
| **Renal conditions** | | 0.03 | | 0.22 | | 0.19 | 0.82 | |
| **METS classification** | | 3.29 | | 2.96 | | 3.64 | 3.17 | |
| **Anaesthetic alert** | | 0.67 | | 0.59 | | 0.43 | 0.60 | |
| **Cardiovascular medications** | | 3.43 | | 4.66 | | 2.92 | 2.82 | |
| **Endocrine medications** | | 2.48 | | 2.84 | | 2.25 | 2.39 | |
| *ICD-10 Code Counts* | |  | |  | |  |  | |
| **II: Cancer conditions** | | 1.70 | | 1.11 | | 1.19 | 0.46 | |
| **IV: Endocrine conditions** | | 1.61 | | 0.94 | | 1.38 | 0.59 | |
| **IX: Cardiovascular conditions** | | 1.34 | | 0.75 | | 1.27 | 0.54 | |
| **VI: Central nervous system conditions** | | 1.17 | | 0.37 | | 1.21 | 0.47 | |
| **XIX: Injury and poisoning conditions** | | 1.0 | | 0.0 | | 1.09 | 0.28 | |
| **XVIII: Other conditions** | | 1.58 | | 0.75 | | 1.32 | 0.72 | |
| **XXII: Other specialized codes** | | 1.24 | | 0.53 | | 1.16 | 0.37 | |
| *ICU demand metrics* | |  | |  | |  |  | |
| **ICU admissions previous day** | | | | | | | | |
| Low demand | | (0) | | (0) | | (0) | (0) | |
| Normal demand | | (81) | | (100) | | (629) | (100) | |
| High demand | | (0) | | (0) | | (0) | (0) | |
| **ICU census previous day** | | | | | | | | |
| Low demand | | (0) | | (0) | | (0) | (0) | |
| Normal demand | | (81) | | (100) | | (629) | (100) | |
| High demand | | (0) | | (0) | | (0) | (0) | |

Table S2: Descriptive statistics for features included in model after feature selection process in the training set. Statistics presented are mean and standard deviation (SD) for continuous variables, and count and percentage of total ICU or non-ICU patients for categorical variables. *Sex was removed during RFE and is not a feature in the final model, but is included here for descriptive purposes. WCC: white cell count, INR: international normalised ratio, METs: metabolic equivalence tasks

## 1.7 Results of experiments to select algorithm, oversampling method, and different feature transformations

Results of experiments performed to select best performing algorithm, oversampling method, and set of options for processing features are shown in sheet ’experiment results valset’ in supplementary material.xlsx. Results shown are performance metrics of models using each combination of options in the validation set.

## 1.8 Recursive Feature Elimination


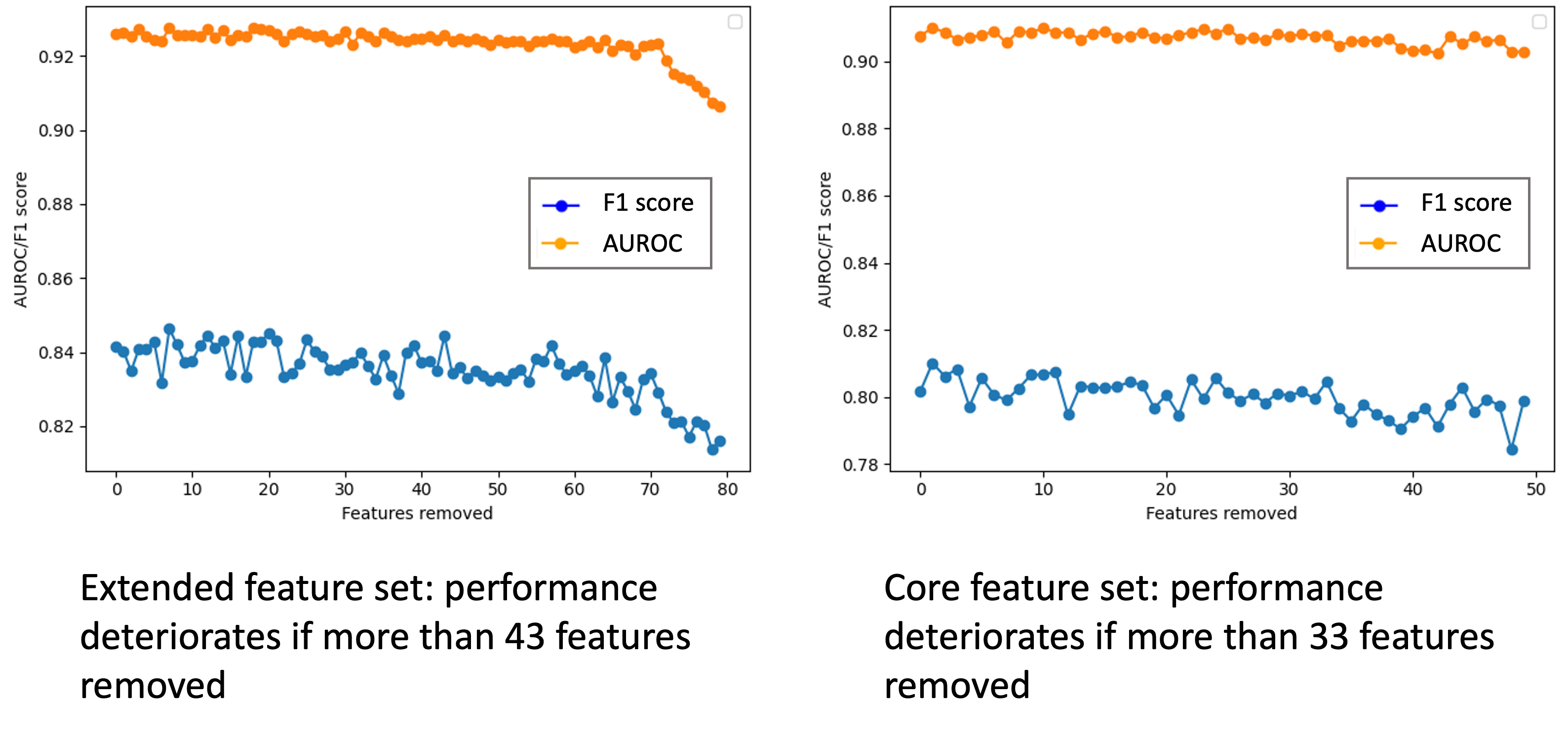


Figure S1: Performance metrics in validation for models generated as features with lowest SHAP value iteratively removed (RFE), showing points at which metrics start to deteriorate

## 1.9 Data completeness in retrospectively dataset and prospective datasets

Data completeness in retrospectively extracted dataset and prospectively extracted dataset is shown in sheet dataset completeness in supplementary material.xlsx. Note, data completeness is not the same as missingness in this context, as some results may only become available in the week prior to surgery, such as an echocardiogram, but would not be treated as missing if absent. The percentage shown for each variable on each number of days prior to surgery is the percentage of cases with data available for that field.

## 1.10 Model performance evaluated in retrospective and prospective test sets


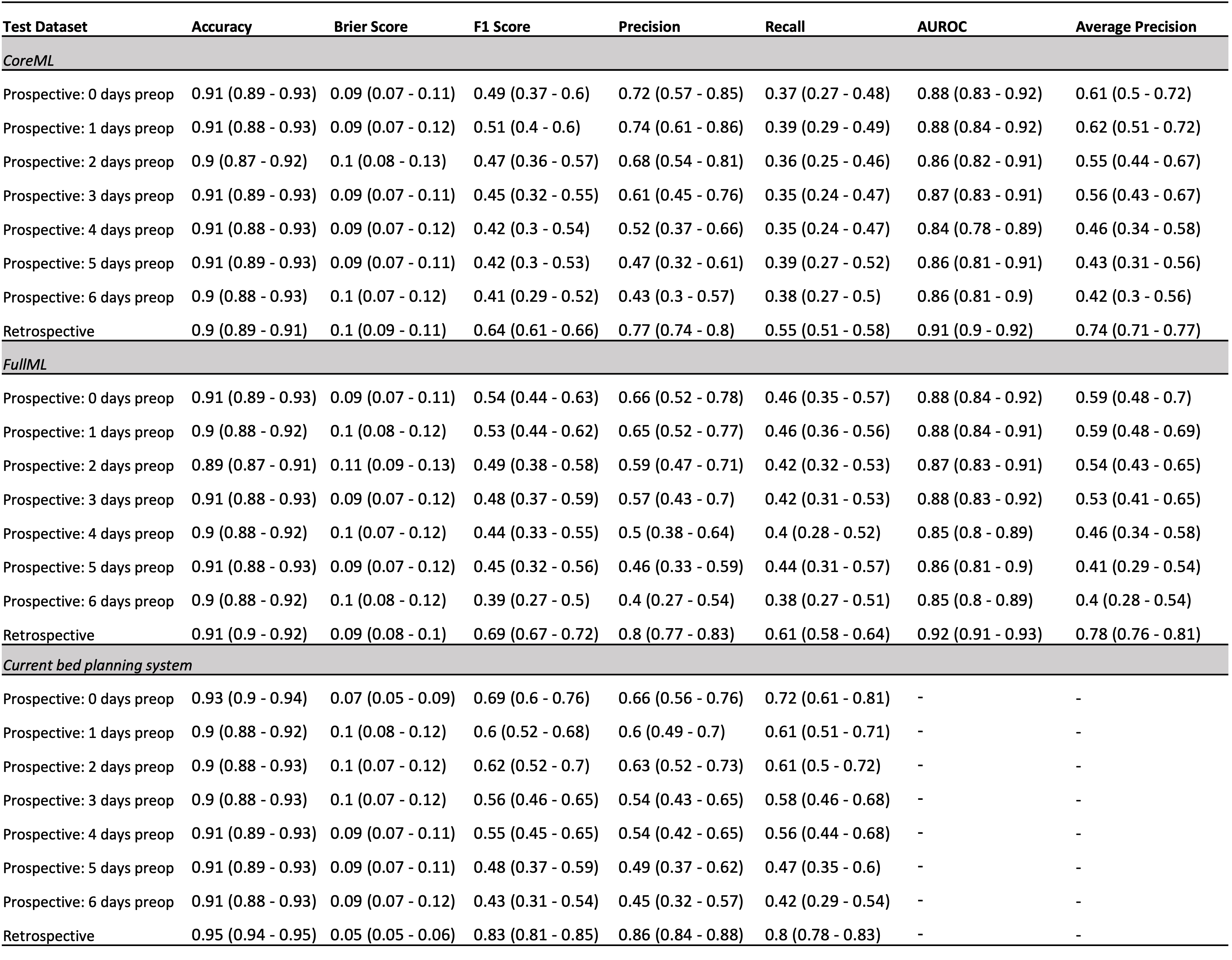


Table S3: Performance of models predicting individual patient level probability of ICU admission. Model performance for CoreML and FullML is shown evaluated in the retrospective test dataset and the prospectively collected dataset with cases partitioned by days until surgery at the time data was extracted from the database


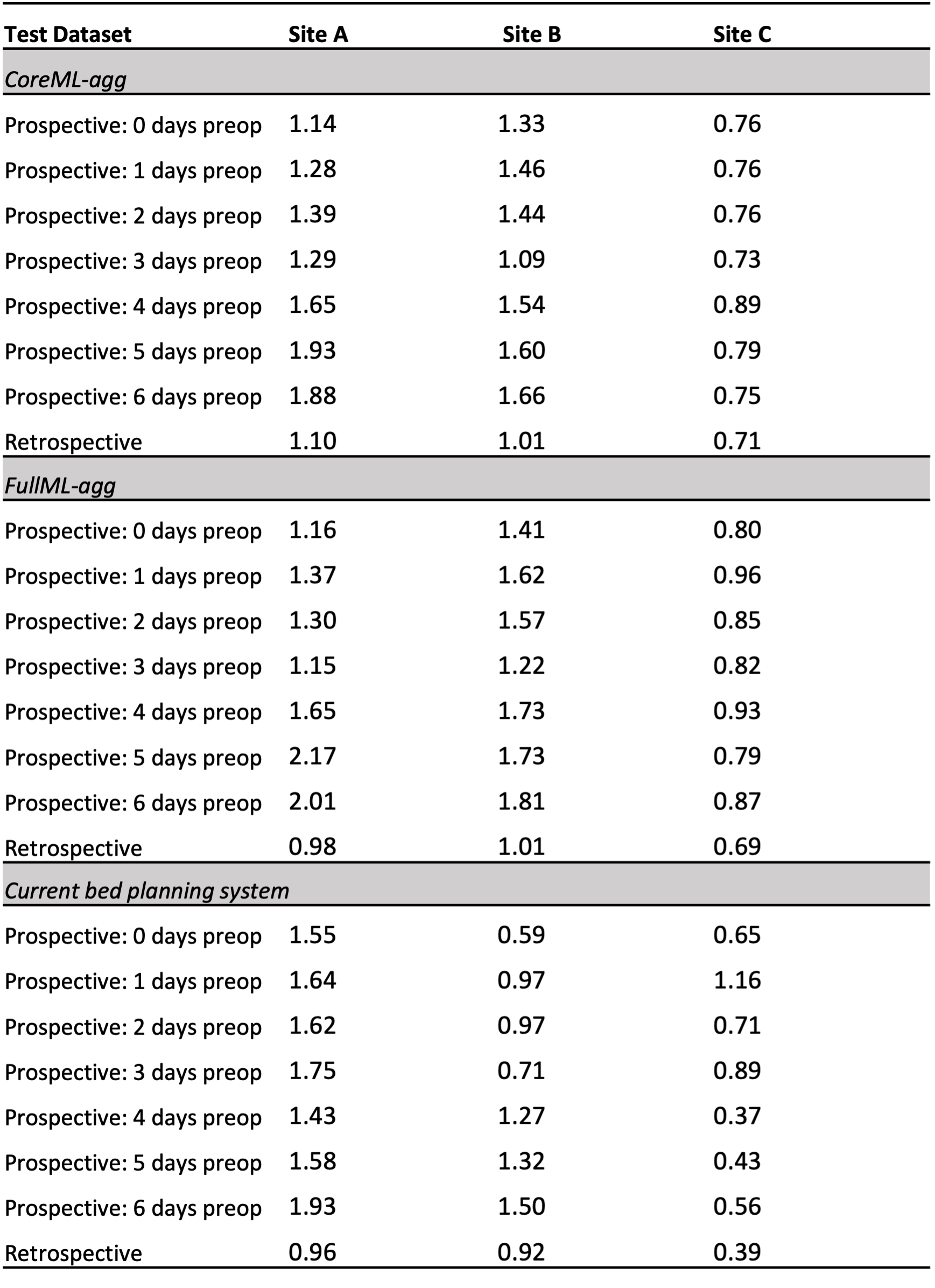


Table S4: Performance of models predicting aggregate performance in predicting daily ICU admissions. Model performance for CoreML and FullML is shown evaluated in the retrospective test dataset and the prospectively collected dataset with cases partitioned by days until surgery at the time data was extracted from the database

## 1.11 Comparison of CoreML model and preassessment postoperative destination aggregate predictions at site B and site C in prospective test dataset


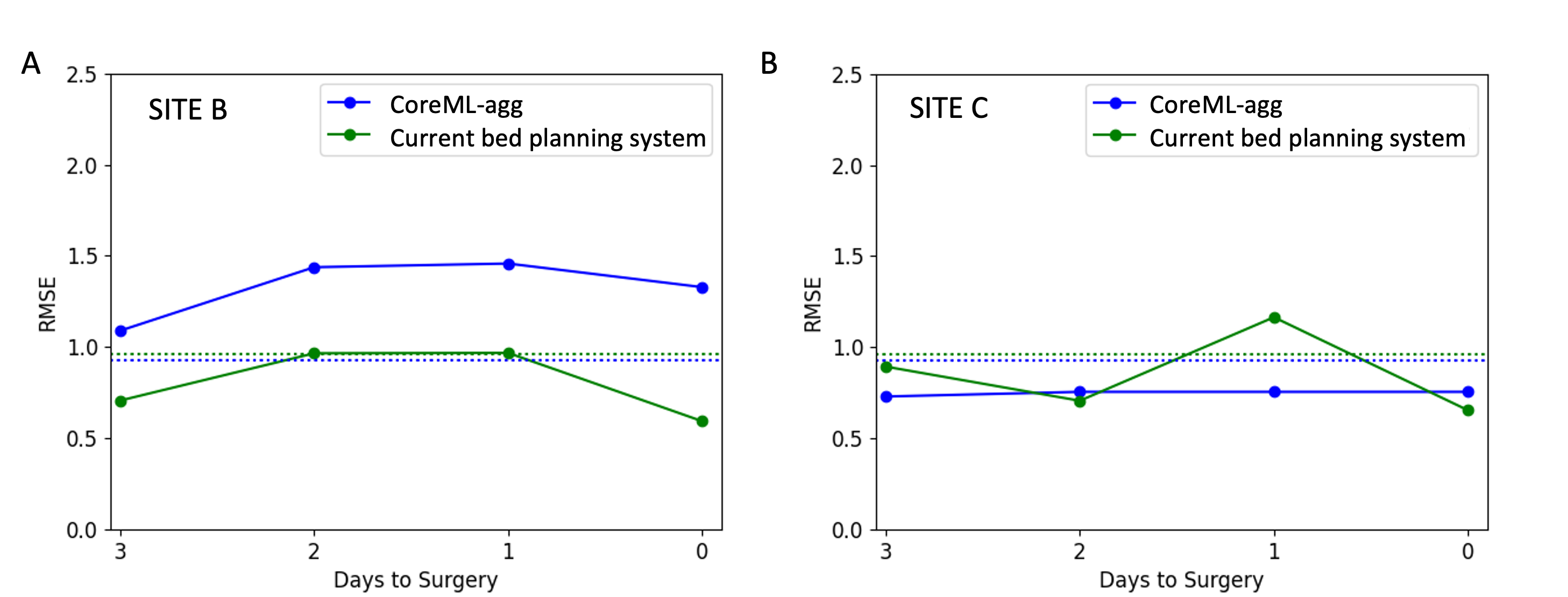


Figure S2: RMSE for core feature set model aggregate predictions and preassessment postoperative destination at sites B and C, evaluated prospectively as predictors of ICU bed demand on data collected over a 3-week period grouped by days prior to surgery at time of prediction. The dotted line of same colour indicates the metric for same predictor in the retrospective test set.

## 1.12 Model performance evaluated in retrospective test set partitioned by month


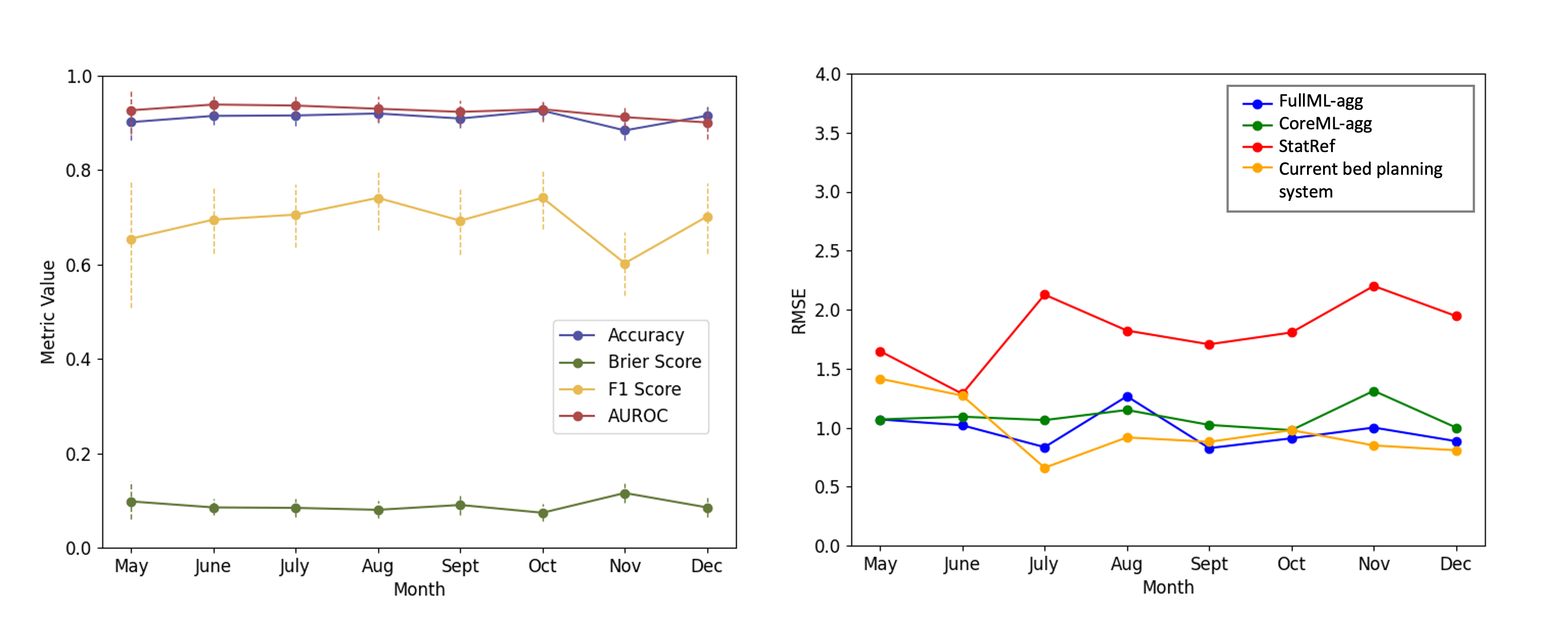


Figure S3: Performance metrics calculated in the retrospective test set partitioned by month, showing change over time to assess for model performance drift

## 1.13 REFORMS checklist

Kapoor et al.^33^.

| **Item Description** | | **Page** | **Notes** |
| --- | --- | --- | --- |
| **Module 1: Study goals** | | | |
| 1a | Population or distribution about which the scientific claim is made. | 7 |  |
| 1b | Motivation for choosing this population or distribution (1a.). | 3 |  |
| 1c | Motivation for the use of ML methods in the study. | 3 |  |
| **Module 2: Computational reproducibility** | | | |
| 2a | Dataset used for training and evaluating the model along with link or DOI to uniquely identify the dataset. |  | Our datasets were extracted from the EHR at UCLH and has not been published separately. |
| 2b | Code used to train and evaluate the model and produce the results reported in the paper along with link or DOI to uniquely identify the version of the code used. |  | Code available on request. |
| 2c | Description of the computing infrastructure used. | 20 |  |
| 2d | README file which contains instructions for generating the results using the provided dataset and code. |  | Code available on request. |
| 2e | Reproduction script to produce all results reported in the paper. |  | Code available on request. |
| **Module 3: Data quality** | | | |
| 3a | Source(s) of data, separately for the training and evaluation datasets (if applicable), along with the time when the dataset(s) are collected, the source and process of ground-truth annotations, and other data documentation. | 4 |  |
| 3b | Distribution or set from which the dataset is sampled (i.e., the sampling frame). | 4 |  |
| 3c | Justification for why the dataset is useful for the modeling task at hand. | 4 |  |
| 3d | The definition of the outcome variable of the model along with descriptive statistics, if applicable. | 7 |  |
| 3e | Number of samples in the dataset. | 7 |  |
| 3f | Percentage of missing data, split by class for a categorical outcome variable. | 26 |  |
| 3g | Justification for why the distribution or set from which the dataset is drawn (3b.) is representative of the one about which the scientific claim is being made (1a.). | 3 |  |
| **Module 4: Data preprocessing** | | | |
| 4a | Identification of whether any samples are excluded with a rationale for why they are excluded. | 4 |  |
| 4b | How impossible or corrupt samples are dealt with. | 21 |  |
| 4c | All transformations of the dataset from its raw form (3a.) to the form used in the model, for instance, treatment of missing data and normalization. | 21 |  |
| **Module 5: Modeling** | | | |
| 5a | Detailed descriptions of all models trained, including: | 4 |  |
|  | All features used in the model (including any feature selection). | 22 |  |
|  | Types of models implemented (e.g., Random Forests, Neural Networks). | 26 |  |
|  | Loss function used. | 21 |  |
| 5b | Justification for the choice of model types implemented. | 21 |  |
| 5c | Method for evaluating the model(s) reported in the paper, including details of train-test splits or cross-validation folds. | 6 |  |
| 5d | Method for selecting the model(s) reported in the paper. | 6 |  |
| 5e | For the model(s) reported in the paper, specify details about the hyperparameter tuning:  Range of hyper-parameters used and a justification for why this range is reasonable. | 22 |  |
|  | Method to select the best hyper-parameter configuration. | 22 |  |
|  | Specification of all hyper-parameters used to generate results reported in the paper. | 22 |  |
| 5f | Justification that model comparisons are against appropriate baselines. | 4 |  |
| **Module 6: Data leakage** | | | |
| 6a | Justification that pre-processing (Section 4) and modeling (Section 5) steps only use information from the training dataset (and not the test dataset). | 21 |  |
| 6b | Methods to address dependencies or duplicates between the training and test datasets (e.g. different samples from the same patients are kept in the same dataset partition). |  | Some patients underwent more than one surgery in the period covered by the retrospective dataset. We have not excluded these cases, as the outcome of whether a patient is admitted to ICU postoperatively is determined by the combination of patient and surgical factors, and each surgical case is unique. The patient’s clinical condition will also have potentially changed between surgeries. No patient identifying features which could allow data leakage were included in the dataset. Our use of a temporal split in our training, validation and testing sets means that the data the model is trained on cannot contain information about the future cases of a patient the model is used to make a prediction for. If the model were to be deployed, it would be the case that a small number of patients present in the training dataset have repeat operations which the model is used to make predictions for, and our reported performance metrics therefore provide a realistic estimate of performance. |
| 6c | Justification that each feature or input used in the model is legitimate for the task at hand and does not lead to leakage. | 21 |  |
| **Module 7: Metrics and uncertainty** | | | |
| 7a | All metrics used to assess and compare model performance (e.g., accuracy, AUROC etc.). Justify that the metric used to select the final model is suitable for the task. | 5 |  |
| 7b | Uncertainty estimates (e.g., confidence intervals, standard deviations), and details of how these are calculated. | 5 |  |
| 7c | Justification for the choice of statistical tests (if used) and a check for the assumptions of the statistical test. |  | No statistical tests used |
| **Module 8: Generalizability and limitations** | | | |
| 8a | Evidence of external validity. |  | We have not performed external validation |
| 8b | Contexts in which the authors do not expect the study’s findings to hold. | 13 |  |
